# Ramadan Fasting and Patients with Cancer: State-of-the-Art and Future Prospects

**DOI:** 10.3389/fonc.2016.00027

**Published:** 2016-02-10

**Authors:** Nicola Luigi Bragazzi, Walid Briki, Hicham Khabbache, Ismail Rammouz, Karim Chamari, Taned Demaj, Tania Simona Re, Mohamed Zouhir

**Affiliations:** ^1^Department of Health Sciences (DISSAL), School of Public Health, University of Genoa, Genoa, Italy; ^2^Section of Psychiatry, Department of Neuroscience, Rehabilitation, Ophthalmology, Genetics, Maternal and Child Health (DINOGMI), University of Genoa, Genoa, Italy; ^3^College of Arts and Sciences, Qatar University, Doha, Qatar; ^4^Laboratoire Etudes théologiques, Sciences Cognitives et Sociales, Faculty of Literature and Humanistic Studies, Sais, Sidi Mohamed Ben Abdellah University, Fez, Morocco; ^5^Psychiatric Centre Ibn Alhassan, CHU Hassan II, Fez, Morocco; ^6^Clinical Neuroscience Laboratory, Sidi Mohamed Ben Abdellah University, Fez, Morocco; ^7^Athlete Health and Performance Research Centre, Aspetar, Qatar Orthopaedic and Sports Medicine Hospital, Doha, Qatar; ^8^Emergency Department (Servizio di Emergenza Sanitaria Territoriale 118), Ospedale Maggiore della Carità, Novara, Italy; ^9^University of Genoa, Genoa, Italy

**Keywords:** cancer, drug compliance and adherence, quality of life, Ramadan fasting, stomia

## Abstract

Ramadan fasting represents one of the five pillars of the Islam creed. Even though some subjects (among which patients) are exempted from observing this religious duty, they may be eager to share this particular moment of the year with their family and peers. However, there are no guidelines or standardized protocols that can help physicians to properly address the issue of patients with cancer fasting in Ramadan and correctly advising them. Moreover, in a more interconnected and globalized society, in which more and more Muslim patients live in the Western countries, this topic is of high interest also for the general practitioner. For this purpose, we carried out a systematic review on the subject. Our main findings are that (1) very few studies have been carried out, addressing this issue, (2) evidence concerning quality of life and compliance to treatment is contrasting and scarce, and (3) generally speaking, few patients ask their physicians whether they can safely fast or not. For these reasons, further research should be performed, given the relevance and importance of this topic.

## Introduction

The Arabic word *Islam* means peace, purity, and total submission to the will of Allah (the Lord of the worlds, *Rabb al-‘alameen*) by conforming inwardly and outwardly to his law. The religion of Islam is based on five pillars (*arkan al-Islam*), and Ramadan fasting is one of these pillars.

In particular, Ramadan fasting represents a particular form of fasting, in that consists of alternate fasting and feasting (re-feeding) periods (1). Being based on the lunar calendar, mean fasting duration varies depending on the period of the year and the latitude of the place (1, 2).

A number of subjects, including patients, are usually exempted from this duty even though they could be willing to fast for taking part into the atmosphere of this particular moment (3).

It is a common saying that religion promotes healthy lifestyles. However, the relationship between a religion and cancer has been often overlooked or poorly addressed from a scientific, rigorous point of view (4), especially in terms of physiology (5). Among the few studies available in the literature, Mills and collaborators investigated Seventh-Day Adventists. This religious group does not consume tobacco, alcohol, or pork and is characterized by lower cancer-related morbidity rates than the general population, even though the risk for prostate and endometrial cancer was higher (6). Fraser noticed that non-vegetarian Seventh-Day Adventists were significantly more affected by colorectal and prostate cancer than vegetarian Seven-Day Adventists (7–9), as well as of meningioma and glioma (10). Seventh-Day Adventists present also a particular relationship between cancer risk and allergy (11). Ogata and coworkers found a lower mortality from respiratory cancers among Zen Buddhists with respect to the general population (12). Sriplung and collaborators found that a lower incidence of cervical and breast cancers in Muslim than in Buddhist females. Furthermore, they noticed a lower incidence of prostate cancer in Muslim males. Independent of sex and year of diagnosis, the incidence of lung, liver, non-Hodgkin lymphoma (NHL), and colorectal cancers was lower in Muslims (13). Islamic beliefs can have an impact on access to breast or cervical cancer screening programs and therefore should be carefully taken into account by health workers in the field of Public Health (14–28).

The effects of Ramadan fasting on patients suffering from cancer interest physicians in that, in the nowadays globalized society, have to face with issues such as the management of cancer in Muslim patients (29).

In principle, clinical investigations carried out among patients suffering from cancer fasting during Ramadan could have broad, translational implications. The effects of fasting on cancer have been extensively studied: starvation-induced changes in genome organization, chromatin remodeling, and metabolic pathways modulating, for example, glucose, insulin-growth factor (IGF), and other related enzymes and proteins may increase the effectiveness of chemotherapy (30–32). Fasting has an anti-Warburg effect and could have a positive impact on drug pharmacokinetics and could contribute to reduce its side effects, improve the chemotherapeutic index, and overcome at least partially the issue of drug resistance (30–32). Furthermore, fasting has a beneficial impact on the renewal of stem cells and on the immune system, in particular on natural killer cells, as well as on the oxidant system, thus enabling cancer growth control (33–36). To this respect, different kinds of cancer have been investigated, such as pancreatic (37), breast (38–40), lung (41), colorectal (42) and prostate (43) cancer, and even glioma (44). The impact of fasting has been assessed on different drugs (45, 46), including irinotecan (47, 48), doxorubicin (49), and on new recent anti-blastics, such as erlotinib (41), tyrosine kinase inhibitors (50), and sirtuin (51), among others.

Effects of fasting on tumor have been assessed both on neo-adjuvant and adjuvant therapy (40). Furthermore, fasting seems to sensitize tumor cells and increase the outcome of radiation therapy (44).

However, despite that this topic has attracted an impressive body of research (52, 53), little is known both in terms of molecular mechanisms and clinical outcomes about the difference among the various existing kinds of fasting, such as periodic diet (54), calories restriction (55), dietary restriction (56) or dietary manipulation (53), intermittent (38, 43, 57) or short-term fasting (40, 49), and prolonged fasting (33, 35, 39), among others. Lv and collaborators in a recently published systematic review and meta-analysis concluded that while the roles of ketogenic diet and of caloric restriction in controlling cancer are quite established, evidence of the effectiveness of intermittent fasting is still insufficient (57). Furthermore, most of these studies have been conducted in animal models; therefore, there is an urgent need for more investigations in human (58).

Ramadan represents a unique laboratory for studying the effect of fasting in cancer patients, using a multidisciplinary approach. Aspects such as diabetic patients (59) or with renal (60, 61), infectious (62), or cardiovascular diseases (63) have been already reviewed.

However, there is a dearth of information concerning cancer patients willing to fast during Ramadan. For this purpose, we have carried out a systematic review, mining different databases, such as ISI/Web of Science (WoS), MEDLINE/PubMed, Scopus, Scirus, Directory of Open Access Journals (DOAJ), and Google Scholar.

## Ramadan and Cancer: Quality of Life and Adherence to Religious Worship

Kuzu and collaborators (29) investigated quality of life and compliance to religious duties (such as praying and fasting) in 178 patients living with a permanent colostomy. Seventy-five underwent abdominoperineal resection (APR, or Miles’ operation), 51 sphincter-saving resection, and 52 anterior resection, including sigmoid colectomy. Quality of life was measured with the Medical Outcomes Study Short Form 36 Health Survey (SF-36) and a questionnaire with items about work, sexual life, and compliance to religious worship. They found that a significantly number of patients in the APR group stopped praying daily (either alone or in a mosque) and fasting during Ramadan. They concluded that colorectal cancer, besides affecting quality of life, has a profound impact on religious worship. The authors recommended a preoperative counseling, involving local religious authorities.

Celasin and coworkers (64) carried out a prospective study recruiting 93 Muslim patients after surgery for colorectal carcinoma: 50 underwent APR, 22 sphincter-saving resection, and 1 anterior resection, including sigmoid colectomy. Quality of life was assessed pre- and postoperatively at 15–18 months with the SF-36 questionnaire and a modified version of the American Society of Colorectal Surgeons (ASCRS) Fecal Incontinence questionnaire. Life standards, including religious practice, were measured using the Ankara University Life Standard Questionnaire (AULSQ). Authors found that religious worship (praying alone, praying in mosques, fasting during Ramadan, and purifying alms) was not significantly different among the groups. Probably, this was the effect of a proper religious counseling and its importance, advocated by Kuzu and collaborators in the previous article (29), is here confirmed.

Zeeneldin and Taha (65) conducted a cross-sectional study during Ramadan, August–September 2009, investigating 102 patients suffering from breast cancer (31%), acute leukemia (24%), colorectal cancer (7%), NHL (5%), bladder cancer (4%), lung cancer (4%), and laryngeal cancer (4%). Treatments included chemotherapy, radiotherapy, hormonal therapy, and non-specific therapy in 42, 31, 10, and 17%, respectively. Comorbidities were present in 22% of the patients. Authors found that 40% of patients did not fast during Ramadan, 36 and 24% were partial and complete fasters. Being female patients, having a good and stable performance status, suffering from a non-metastatic solid tumor, and receiving non-intravenous chemotherapy as outpatients were found to be predictor of compliance to fasting. However, only 46% of patients sought the treating oncologist advice on whether they could fast.

Altuntas and coworkers (66) carried out a prospective study investigating 56 patients with a cancer-related fecal stoma over two periods of Ramadan to analyze the effect of fasting on nutritional and metabolic status and quality of life. Fourteen patients were fasting. They had their stoma for a longer period of time than patients in the non-fasting group, and the proportion of patients with a permanent stoma was higher in the fasting group than in the non-fasting group. Ramadan fasting had almost no influence on quality of life.

Tas and collaborators (67) performed a survey of 701 adult Turkish Muslim cancer patients during the month of Ramadan in 2012. Before diagnosis of cancer, 93.1% of the patients used to fast completely or partially. After diagnosis of cancer, this rate fell down to 15%: 83.9% of patients who fasted before diagnosis, gave up observing the religious duty. Patients who decided to go on fasting after the diagnosis of cancer thought that observing Ramadan would have lead to Patients who were females, those with good performance status, those without any comorbid disease (in particular, diabetes mellitus), who had non-metastatic disease, those with history of surgery, young, those treated with radiotherapy, and those being treated with single agents, oral chemotherapeutic agents, or not receiving drugs being in the follow-up period were more likely to be fasting than others. The fasting ones suffered from lymphoma, urogenital cancer (in particular, testicular tumor), and breast cancer; conversely, the rate of fasting status among patients with lung and gastrointestinal cancer was quite low. Gynecologic, head and neck, sarcoma, and skin cancers did not correlate with fasting status. Only 20.8% of all patients asked their physician whether it was alright for them to fast and physicians generally had a negative attitude toward fasting (83.2%). About 13.3% of physicians allowed patients to choose whether to fast or not. Physicians were concerned about the possibility of fasting in patients at risk of tumor lysis syndrome, taking nephrotoxic drugs, or other drugs that could lead to vomiting, diarrhea, or renal failure. The authors concluded that majority of cancer patients are not fasting during the month of Ramadan, and a small part of patients consult this situation to their physician.

## Ramadan and Cancer: Adherence to Drug Treatment

Drug compliance during Ramadan generally tends to fall, as noticed by some scholars (68–70).

Other authors, such as Zeeneldin and coauthors (71), however, have found that Ramadan fasting does not impair patient’s adherence to treatment. During Ramadan 2010, 139 patients suffering from breast cancer were asked about compliance to fasting and religious duties as well as to treatment with oral hormonal therapy (OHT) in Ramadan and in the preceding month. Tamoxifen and aromatase inhibitors were used in 64 and 36%, respectively. Adherence rates to OHT during Ramadan and before were 94.2 and 95.7%, respectively (not statistically significant). Non-adherence prior to Ramadan and shorter duration of OHT were predictors of non-adherence during Ramadan, while fasting status, age, performance status, presence of metastases, and type of OHT were not good predictors of adherence.

## Results and Discussion

Despite our extensive search, we managed to find only six studies addressing the issue of patients suffering from cancer fasting during Ramadan: in particular, five focusing on the impact of fasting on quality of life and compliance to worship and one focusing on adherence to drug treatment. Despite the importance and relevance of this topic and given the burden imposed by the cancer, very few scholars have investigated the impact of Ramadan fasting on the health of patients with tumor.

The effect of Ramadan on quality of life appears to be controversial: while three studies confirm this relationship, two other researches fail to find any impact. Also, the effect of fasting on compliance to worship seems to be quite contrasting.

As far as the compliance to drug treatment is concerned, there is only one study available, and therefore it is not possible to collect any evidence.

## Conclusion

There is a strong need for evidence-based suggestions and guidelines (3). Very few studies are available, with contrasting findings.

The management of Muslim patients suffering from cancer is very complex and should involve a multidisciplinary team, made up of an oncologist, a nutritionist, a psychiatrist, or a psychologist, who should be aware of the importance of cultural and spiritual beliefs in medicine (5), and in particular in the oncology practice (72).

A diagnosis of cancer is always difficult to cope with and in Muslim subjects can have a varying impact, depending also on the degree of religiosity of the subjects itself. Errihani and colleagues found that in practicing Muslims cancer is seen as a divine test and as such accepted, while non-practicing Muslims feel guilty and begin to practice (73). Therefore, the level of spirituality/religiosity plays a major role in supporting the patient during the health-care process and can influence the clinical outcome. However, as found by the study of Tas and collaborators (67), the communication between the patient and the physician is not always characterized by an open disclosure and a frank dialog. This is further complicated by the fact that the situation is rather delicate and the doctor, if not culturally competent or sensitive, could be left with dilemmas because of the cultural/religious beliefs and attitudes of the patient (74).

Oncologists should carefully assess the health conditions of patients and discourage them to fast if they present metastasis or particularly disseminated, aggressive forms of cancer or if they have a history of non-compliance/non-adherence to pharmacological and/or dietary treatment.

On the contrary, patients with a strong motivation to fast should be encourage, in that spirituality/religiosity plays a major role in cancer. The patient feels indeed himself/herself more active being involved in the religious activities, and less depressed and isolated (3). Furthermore, there is a solid scientific evidence that fasting, activating certain neuroendocrine pathways and leading to the release of neurotrophic factors, can enhance the mood and relieve the pain in patients suffering from chronic disorders (75).

A particular aspect that should be addressed is the management of terminal patients during Ramadan, especially in terms of terminal nutrition (76). From the literature, it is known that some Muslim patients with terminal illnesses may express a strong spiritual and religious wish (77, 78). This calls for cooperation between the family and the interdisciplinary palliative care team. On the contrary, some scholars, like Tas and collaborators (67), have shown that there is poor cooperation between physician and patient. Facing patients’ needs is likely to have a great positive impact on the patient’s sense of wellbeing.

However, once again, also this topic has been poorly explored, despite the clinical importance of fasting (79). Therefore, further research in the field is needed.

## Author Contributions

All authors conceived the design of the study and wrote the paper.

## Conflict of Interest Statement

The authors declare that the research was conducted in the absence of any commercial or financial relationships that could be construed as a potential conflict of interest.

## References

[1] Berbari AE Daouk NA Mallat SG Jurjus AR Ramadan fasting in health and disease. In: Berbari AE Mancia G, editors. Special Issues in Hypertension. Milan: Springer-Verlag (2012). p. 331–46.

[2] Günaydin GP Dogan NO Çevik Y Korkmaz H Savrun A Çıkrıkçı G Evaluation of patients with renal colic that present to an emergency department during the month of Ramadan. [Ramazan Ayinda Renal Kolikle Acil Servise Basvuran Hastalarin Degerlendirilmesi]. Acad Emerg Med (2013) 12:24–6. 10.5152/jaem.2012.032

[3] Al Wakeel J Mitwalli AH Alsuwaida A Al Ghonaim M Usama S Hayat A Recommendations for fasting in Ramadan for patients on peritoneal dialysis. Perit Dial Int (2013) 33:86–91. 10.3747/pdi.2010.00095 23349195 PMC3598257

[4] Sarri KO Higgins S Kafatos AG Are religions “healthy”? A review of religious recommendations on diet and lifestyle. Ecol Cult Nutr Health Dis (2006):7–20.

[5] Holt-Lunstad J Steffen PR Sandberg J Jensen B. Understanding the connection between spiritual well-being and physical health: an examination of ambulatory blood pressure, inflammation, blood lipids and fasting glucose. J Behav Med (2011) 34(6):477–88. 10.1007/s10865-011-9343-7 21487720

[6] Fraser GE. Associations between diet and cancer, ischemic heart disease, and all-cause mortality in non-Hispanic White California Seventh-Day Adventists. Am J Clin Nutr (1999) 70(3 Suppl):532S–8S. 10479227 10.1093/ajcn/70.3.532s

[7] Mills PK Beeson WL Phillips RL Fraser GE. Cancer incidence among California Seventh-Day Adventists, 1976-1982. Am J Clin Nutr (1994) 59(5 Suppl):1136S–42S. 8172114 10.1093/ajcn/59.5.1136S

[8] Mills PK Beeson WL Phillips RL Fraser GE. Bladder cancer in a low risk population: results from the Adventist Health Study. Am J Epidemiol (1991) 133(3):230–9. 2000840 10.1093/oxfordjournals.aje.a115867

[9] Beeson WL Mills PK Phillips RL Andress M Fraser GE. Chronic disease among Seventh-Day Adventists, a low-risk group. Rationale, methodology, and description of the population. Cancer (1989) 64(3):570–81. 10.1002/1097-0142(19890801)64:3<570::AID-CNCR2820640303>3.0.CO;2-4 2743251

[10] Mills PK Preston-Martin S Annegers JF Beeson WL Phillips RL Fraser GE. Risk factors for tumors of the brain and cranial meninges in Seventh-Day Adventists. Neuroepidemiology (1989) 8(5):266–75. 10.1159/000110193 2812186

[11] Mills PK Beeson WL Fraser GE Phillips RL. Allergy and cancer: organ site-specific results from the Adventist Health Study. Am J Epidemiol (1992) 136(3):287–95. 1415150 10.1093/oxfordjournals.aje.a116494

[12] Ogata M Ikeda M Kuratsune M. Mortality among Japanese Zen priests. J Epidemiol Community Health (1984) 38(2):161–6. 10.1136/jech.38.2.161 6747517 PMC1052341

[13] Sriplung H Bilheem S Kuntipundee T Geater SL. Differences in cancer incidence among predominantly Muslim and Buddhist subpopulations in Songkhla. Asian Pac J Cancer Prev (2014) 15(22):9979–83. 10.7314/APJCP.2014.15.22.9979 25520139

[14] Khan S Woolhead G. Perspectives on cervical cancer screening among educated Muslim women in Dubai (the UAE): a qualitative study. BMC Womens Health (2015) 15(1):90. 10.1186/s12905-015-0252-8 26496942 PMC4619445

[15] Hasnain M Menon U Ferrans CE Szalacha L. Breast cancer screening practices among first-generation immigrant Muslim women. J Womens Health (Larchmt) (2014) 23(7):602–12. 10.1089/jwh.2013.4569 24865517 PMC4089017

[16] Salad J Verdonk P de Boer F Abma TA. “A Somali girl is Muslim and does not have premarital sex. Is vaccination really necessary?” A qualitative study into the perceptions of Somali women in the Netherlands about the prevention of cervical cancer. Int J Equity Health (2015) 14:68. 10.1186/s12939-015-0198-3 26293806 PMC4546144

[17] Hatefnia E Niknami S Bazargan M Mahmoodi M Lamyianm M Alavi N. Correlates of mammography utilization among working Muslim Iranian women. Health Care Women Int (2010) 31(6):499–514. 10.1080/07399331003725507 20461601

[18] Matin M LeBaron S. Attitudes toward cervical cancer screening among Muslim women: a pilot study. Women Health (2004) 39(3):63–77. 10.1300/J013v39n03_05 15256356

[19] Underwood SM Shaikha L Bakr D. Veiled yet vulnerable. Breast cancer screening and the Muslim way of life. Cancer Pract (1999) 7(6):285–90. 10.1046/j.1523-5394.1999.76004.x 10732525

[20] Alagraa R Abujaber A Chandra P Doughty J. Evaluating psychosocial support needs of female cancer patients in the State of Qatar. Qatar Med J (2015) 2015(1):4. 10.5339/qmj.2015.4 26535172 PMC4614333

[21] Rasul S Khan KS Rizvi JH Hassan SH Maniar S. Cervical cancer screening program in a Muslim country: three-year experience at the Aga Khan University Medical Center, Karachi. Asia Oceania J Obstet Gynaecol (1991) 17(1):1–4. 10.1111/j.1447-0756.1991.tb00243.x 2064585

[22] Al-Amoudi S Cañas J Hohl SD Distelhorst SR Thompson B. Breaking the silence: breast cancer knowledge and beliefs among Somali Muslim women in Seattle, Washington. Health Care Women Int (2015) 36(5):608–16. 10.1080/07399332.2013.857323 24351062 PMC8856570

[23] Badar F Anwar N Meerza F Sultan F Cervical carcinoma in a Muslim community. Asian Pac J Cancer Prev (2007) 8(1):24–6. 17477766

[24] Harandy TF Ghofranipour F Montazeri A Anoosheh M Bazargan M Mohammadi E Muslim breast cancer survivor spirituality: coping strategy or health seeking behavior hindrance? Health Care Women Int (2010) 31(1):88–98. 10.1080/07399330903104516 20390638

[25] Banning M Hafeez H. Perceptions of breast health practices in Pakistani Muslim women. Asian Pac J Cancer Prev (2009) 10(5):841–7. 20104976

[26] Rajaram SS Rashidi A. Asian-Islamic women and breast cancer screening: a socio-cultural analysis. Women Health (1999) 28(3):45–58. 10.1300/J013v28n03_04 10374807

[27] Szarewski A Cadman L Ashdown-Barr L Waller J. Exploring the acceptability of two self-sampling devices for human papillomavirus testing in the cervical screening context: a qualitative study of Muslim women in London. J Med Screen (2009) 16(4):193–8. 10.1258/jms.2009.009069 20054094

[28] Mijit F Ablimit T Abduxkur G Abliz G. Distribution of human papillomavirus (HPV) genotypes detected by routine pap smear in Uyghur-Muslim women from Karasay Township Hotan (Xinjiang, China). J Med Virol (2015) 87(11):1960–5. 10.1002/jmv.24240 26081269 PMC5033003

[29] Kuzu MA Topçu O Uçar K Ulukent S Unal E Erverdi N Effect of sphincter-sacrificing surgery for rectal carcinoma on quality of life in Muslim patients. Dis Colon Rectum (2002) 45(10):1359–66. 10.1007/s10350-004-6425-4 12394435

[30] Goldstein I Hager GL. Transcriptional and chromatin regulation during fasting – the genomic era. Trends Endocrinol Metab (2015) 26(12):699–710. 10.1016/j.tem.2015.09.005 26520657 PMC4673016

[31] Lee C Raffaghello L Longo VD. Starvation, detoxification, and multidrug resistance in cancer therapy. Drug Resist Updat (2012) 15(1–2):114–22. 10.1016/j.drup.2012.01.004 22391012 PMC3573885

[32] Lee C Safdie FM Raffaghello L Wei M Madia F Parrella E Reduced levels of IGF-I mediate differential protection of normal and cancer cells in response to fasting and improve chemotherapeutic index. Cancer Res (2010) 70(4):1564–72. 10.1158/0008-5472.CAN-09-3228 20145127 PMC2836202

[33] Cheng CW Adams GB Perin L Wei M Zhou X Lam BS Prolonged fasting reduces IGF-1/PKA to promote hematopoietic-stem-cell-based regeneration and reverse immunosuppression. Cell Stem Cell (2014) 14(6):810–23. 10.1016/j.stem.2014.04.014 24905167 PMC4102383

[34] Chen X Lin X Li M. Comprehensive modulation of tumor progression and regression with periodic fasting and refeeding circles via boosting IGFBP-3 loops and NK responses. Endocrinology (2012) 153(10):4622–32. 10.1210/en.2011-2101 22903617

[35] Mendelsohn AR Larrick JW. Prolonged fasting/refeeding promotes hematopoietic stem cell regeneration and rejuvenation. Rejuvenation Res (2014) 17(4):385–9. 10.1089/rej.2014.1595 25072352

[36] Jakobs HH Mikula M Havemeyer A Strzalkowska A Borowa-Chmielak M Dzwonek A The N-reductive system composed of mitochondrial amidoxime reducing component (mARC), cytochrome b5 (CYB5B) and cytochrome b5 reductase (CYB5R) is regulated by fasting and high fat diet in mice. PLoS One (2014) 9(8):e105371. 10.1371/journal.pone.0105371 25144769 PMC4140751

[37] D’Aronzo M Vinciguerra M Mazza T Panebianco C Saracino C Pereira SP Fasting cycles potentiate the efficacy of gemcitabine treatment in in vitro and in vivo pancreatic cancer models. Oncotarget (2015) 6(21):18545–57. 10.18632/oncotarget.4186 26176887 PMC4621909

[38] Lankelma J Kooi B Krab K Dorsman JC Joenje H Westerhoff HV. A reason for intermittent fasting to suppress the awakening of dormant breast tumors. Biosystems (2015) 127:1–6. 10.1016/j.biosystems.2014.11.001 25448890

[39] Marinac CR Natarajan L Sears DD Gallo LC Hartman SJ Arredondo E Prolonged nightly fasting and breast cancer risk: findings from NHANES (2009-2010). Cancer Epidemiol Biomarkers Prev (2015) 24(5):783–9. 10.1158/1055-9965.EPI-14-1292 25896523 PMC4417458

[40] de Groot S Vreeswijk MP Welters MJ Gravesteijn G Boei JJ Jochems A The effects of short-term fasting on tolerance to (neo) adjuvant chemotherapy in HER2-negative breast cancer patients: a randomized pilot study. BMC Cancer (2015) 15:652. 10.1186/s12885-015-1663-5 26438237 PMC4595051

[41] Katsuya Y Fujiwara Y Sunami K Utsumi H Goto Y Kanda S Comparison of the pharmacokinetics of erlotinib administered in complete fasting and 2 h after a meal in patients with lung cancer. Cancer Chemother Pharmacol (2015) 76(1):125–32. 10.1007/s00280-015-2778-8 25994853

[42] Bianchi G Martella R Ravera S Marini C Capitanio S Orengo A Fasting induces anti-Warburg effect that increases respiration but reduces ATP-synthesis to promote apoptosis in colon cancer models. Oncotarget (2015) 6(14):11806–19. 10.18632/oncotarget.3688 25909219 PMC4494906

[43] Thomas JA II Antonelli JA Lloyd JC Masko EM Poulton SH Phillips TE Effect of intermittent fasting on prostate cancer tumor growth in a mouse model. Prostate Cancer Prostatic Dis (2010) 13(4):350–5. 10.1038/pcan.2010.24 20733612

[44] Safdie F Brandhorst S Wei M Wang W Lee C Hwang S Fasting enhances the response of glioma to chemo- and radiotherapy. PLoS One (2012) 7(9):e44603. 10.1371/journal.pone.0044603 22984531 PMC3439413

[45] Lee C Raffaghello L Brandhorst S Safdie FM Bianchi G Martin-Montalvo A Fasting cycles retard growth of tumors and sensitize a range of cancer cell types to chemotherapy. Sci Transl Med (2012) 4(124):124ra27. 10.1126/scitranslmed.3003293 22323820 PMC3608686

[46] Raffaghello L Safdie F Bianchi G Dorff T Fontana L Longo VD. Fasting and differential chemotherapy protection in patients. Cell Cycle (2010) 9(22):4474–6. 10.4161/cc.9.22.13954 21088487 PMC3048045

[47] Huisman SA Bijman-Lagcher W IJzermans JN Smits R de Bruin RW. Fasting protects against the side effects of irinotecan but preserves its anti-tumor effect in Apc15lox mutant mice. Cell Cycle (2015) 14(14):2333–9. 10.1080/15384101.2015.1044170 25955194 PMC4613178

[48] Huisman SA de Bruijn P Ghobadi Moghaddam-Helmantel IM IJzermans JN Wiemer EA Mathijssen RH Fasting protects against the side-effects of irinotecan treatment but does not abrogate anti-tumor activity in mice. Br J Pharmacol (2015). 10.1111/bph.13317 26332723 PMC4761088

[49] Dirks-Naylor AJ Kouzi SA Yang S Tran NT Bero JD Mabolo R Can short-term fasting protect against doxorubicin-induced cardiotoxicity? World J Biol Chem (2014) 5(3):269–74. 10.4331/wjbc.v5.i3.269 25225594 PMC4160520

[50] Caffa I D’Agostino V Damonte P Soncini D Cea M Monacelli F Fasting potentiates the anticancer activity of tyrosine kinase inhibitors by strengthening MAPK signaling inhibition. Oncotarget (2015) 6(14):11820–32. 10.18632/oncotarget.3689 25909220 PMC4494907

[51] Zhu Y Yan Y Gius DR Vassilopoulos A. Metabolic regulation of Sirtuins upon fasting and the implication for cancer. Curr Opin Oncol (2013) 25(6):630–6. 10.1097/01.cco.0000432527.49984.a3 24048020 PMC5525320

[52] Michalsen A Li C. Fasting therapy for treating and preventing disease – current state of evidence. Forsch Komplementmed (2013) 20(6):444–53. 10.1159/000357765 24434759

[53] Simone BA Champ CE Rosenberg AL Berger AC Monti DA Dicker AP Selectively starving cancer cells through dietary manipulation: methods and clinical implications. Future Oncol (2013) 9(7):959–76. 10.2217/fon.13.31 23837760

[54] Brandhorst S Choi IY Wei M Cheng CW Sedrakyan S Navarrete G A periodic diet that mimics fasting promotes multi-system regeneration, enhanced cognitive performance, and healthspan. Cell Metab (2015) 22(1):86–99. 10.1016/j.cmet.2015.05.012 26094889 PMC4509734

[55] Eslami S Barzgari Z Saliani N Saeedi N Barzegari A. Annual fasting; the early calories restriction for cancer prevention. Bioimpacts (2012) 2(4):213–5. 10.5681/bi.2012.028 23678462 PMC3648937

[56] Lee C Longo VD. Fasting vs dietary restriction in cellular protection and cancer treatment: from model organisms to patients. Oncogene (2011) 30(30):3305–16. 10.1038/onc.2011.91 21516129

[57] Lv M Zhu X Wang H Wang F Guan W. Roles of caloric restriction, ketogenic diet and intermittent fasting during initiation, progression and metastasis of cancer in animal models: a systematic review and meta-analysis. PLoS One (2014) 9(12):e115147. 10.1371/journal.pone.0115147 25502434 PMC4263749

[58] Safdie FM Dorff T Quinn D Fontana L Wei M Lee C Fasting and cancer treatment in humans: a case series report. Aging (Albany NY) (2009) 1(12):988–1007. 20157582 10.18632/aging.100114 PMC2815756

[59] Vasan SK Karol R Mahendri NV Arulappan N Jacob JJ Thomas N. A prospective assessment of dietary patterns in Muslim subjects with type 2 diabetes who undertake fasting during Ramadan. Indian J Endocrinol Metab (2012) 16(4):552–7. 10.4103/2230-8210.98009 22837915 PMC3401755

[60] Bragazzi NL. Ramadan fasting and chronic kidney disease: does estimated glomerular filtration rate change after and before Ramadan? Insights from a mini meta-analysis. Int J Nephrol Renovasc Dis (2015) 8:53–7. 10.2147/IJNRD.S61718 26082658 PMC4459622

[61] Bragazzi NL. Ramadan fasting and chronic kidney disease: a systematic review. J Res Med Sci (2014) 19(7):665–76. 25364369 PMC4214028

[62] Bragazzi NL Briki W Khabbache H Rammouz I Mnadla S Demaj T Ramadan fasting and infectious diseases: a systematic review. J Infect Dev Ctries (2015) 9(11):1186–94. 10.3855/jidc.5815 26623627

[63] Salim I Al Suwaidi J Ghadban W Alkilani H Salam AM. Impact of religious Ramadan fasting on cardiovascular disease: a systematic review of the literature. Curr Med Res Opin (2013) 29(4):343–54. 10.1185/03007995.2013.774270 23391328

[64] Celasin H Karakoyun R Yılmaz S Elhan AH Erkek B Kuzu MA. Quality of life measures in Islamic rectal carcinoma patients receiving counselling. Colorectal Dis (2011) 13(7):e170–5. 10.1111/j.1463-1318.2011.02649.x 21651692

[65] Zeeneldin AA Taha FM. Fasting among Muslim cancer patients during the holy month of Ramadan. Ann Saudi Med (2012) 32(3):243–9. 10.5144/0256-4947.2012.243 22588434 PMC6081034

[66] Altuntas YE Gezen FC Sahoniz T Kement M Aydin H Sahin F Ramadan fasting in patients with a stoma: a prospective study of quality of life and nutritional status. Ostomy Wound Manage (2013) 59(5):26–32. 23669258

[67] Tas F Karabulut S Ciftci R Yildiz I Keskin S Kilic L The behavior of Turkish cancer patients in fasting during the holy month of Ramadan. Jpn J Clin Oncol (2014) 44(8):705–10. 10.1093/jjco/hyu070 24868079

[68] Celik I Barista I Firat D Cancer therapy during Ramadan. J Natl Cancer Inst (1996) 88(12):838 10.1093/jnci/88.12.838 8637051

[69] Patel T Magdum A Ghura V. Does fasting during Ramadan affect the use of topical dermatological treatment by Muslim patients in the UK? Clin Exp Dermatol (2012) 37(7):718–21. 10.1111/j.1365-2230.2012.04403.x 22681415

[70] Yakasai AM Muhammad H Babashani M Jumare J Abdulmumini M Habib AG. Once-daily antiretroviral therapy among treatment-experienced Muslim patients fasting for the month of Ramadan. Trop Doct (2011) 41(4):233–5. 10.1258/td.2011.110130 21914677

[71] Zeeneldin AA Gaber AA Taha FM. Does fasting during Ramadan trigger non-adherence to oral hormonal therapy in breast cancer patients? J Egypt Natl Canc Inst (2012) 24(3):133–7. 10.1016/j.jnci.2012.06.003 22929919

[72] Ong KJ Back MF Lu JJ Shakespeare TS Wynne CJ. Cultural attitudes to cancer management in traditional South-East Asian patients. Australas Radiol (2002) 46(4):370–4. 10.1046/j.1440-1673.2002.t01-1-01085.x 12452906

[73] Errihani H Mrabti H Boutayeb S El Ghissassi I El Mesbahi O Hammoudi M Impact of cancer on Moslem patients in Morocco. Psychooncology (2008) 17(1):98–100. 10.1002/pon.1200 17458922

[74] Khan Khattak MMA Abu Bakar I Yeim L Does religious fasting increase fat free mass (FFM) and reduce abdominal obesity? Nutr Food Sci (2012) 42(2):87–96. 10.1108/00346651211212042

[75] Michalsen A. Prolonged fasting as a method of mood enhancement in chronic pain syndromes: a review of clinical evidence and mechanisms. Curr Pain Headache Rep (2010) 14(2):80–7. 10.1007/s11916-010-0104-z 20425196

[76] Winter SM. Terminal nutrition: framing the debate for the withdrawal of nutritional support in terminally ill patients. Am J Med (2000) 109(9):723–6. 10.1016/S0002-9343(00)00609-4 11137488

[77] Tazi I Ramadan and cancer. J Clin Oncol (2008) 26(33):5485 10.1200/JCO.2008.19.7541 18936464

[78] Whyte A. Ready for Ramadan. Nurs Stand (2010) 24(44):18–9. 10.7748/ns2010.03.24.27.18.w38003 20687282

[79] Longo VD Mattson MP. Fasting: molecular mechanisms and clinical applications. Cell Metab (2014) 19(2):181–92. 10.1016/j.cmet.2013.12.008 24440038 PMC3946160

